# Which lecturers’ characteristics facilitate the learning process? A qualitative study on students’ perceptions in the rehabilitation sciences

**DOI:** 10.1186/s12909-023-04308-y

**Published:** 2023-06-12

**Authors:** Simone Battista, Laura Furri, Valeria Pellegrini, Benedetto Giardulli, Ilaria Coppola, Marco Testa, Andrea Dell’Isola

**Affiliations:** 1 Department of Neurosciences, Rehabilitation, Ophthalmology, Genetics, Maternal and Child Health, University of Genoa, Genoa, Italy; 2 Clinical Epidemiology Unit, Department of Clinical Sciences, Lund University, Orthopaedics, Lund, Wigerthuset, Remissgatan Sweden; 3 School of Medicine and Surgery, University of Verona, Verona, 37135 Italy; 4 Department of Education Sciences, School of Social Sciences, University of Genoa, Genoa, Italy

**Keywords:** Teaching, Education, Social Identification, Health Personnel, Rehabilitation

## Abstract

**Background:**

In education, lecturers play a crucial role in facilitating students’ learning process. However, only a few studies explored which lecturers’ characteristics can facilitate this process in higher education for rehabilitation healthcare professionals. Starting from students’ perspectives, our qualitative study investigated the lecturers’ characteristics that facilitate students’ learning process in the rehabilitation sciences.

**Methods:**

A qualitative interview study. We enrolled students attending the 2nd year of the Master of Science (MSc) degree in ‘Rehabilitation Sciences of Healthcare Professions’. Different themes were generated following a ‘Reflexive Thematic Analysis’.

**Results:**

Thirteen students completed the interviews. From their analysis, we generated five themes. Specifically, a lecturer that facilitates students’ learning process should be: 1) ‘A Performer who Interacts with the Classroom’, 2) A Flexible Planner who Adopts Innovative Teaching Skills’, 3) ‘A Motivator who Embraces Transformational Leadership’, 4) ‘A Facilitator Who Encourages a Constructive Learning Context’ and 5) ‘A Coach who Devises Strategies to Reach Shared Learning Goals’.

**Conclusions:**

The results of this study underscore the importance for lecturers in rehabilitation to cultivate a diverse set of skills drawn from the arts and performance, education, team building and leadership to facilitate students’ learning process. By developing these skills, lecturers can design lessons that are worth attending not only for their relevant content but also for their value in human experience.

## Introduction

Performance-based metrics are becoming paramount to assessing the quality of education and institutions [1, 2]. To reach optimal levels of these metrics, there has been a blooming interest in studying lecturers’ professional development [3] as teaching is one of the most critical aspects of students’ learning experiences [4–6]. Nevertheless, evidence on lecturers’ development rarely addresses which characteristics they should develop [3] and considers students’ perceptions. Furthermore, the concept of ‘active learning’ is becoming vital in higher education. In this new learning paradigm, the lecturer facilitates students’ learning process by building knowledge with them rather than merely transmitting it [7]. The lecturer-student relationship is predominant in this new learning paradigm, and lecturers’ interpersonal skills, attitudes and characteristics assume even greater relevance [8]. Therefore, it is essential to argue which lecturers’ characteristics facilitate students’ learning process.

Two recent reviews by Chuenjitwongsa et al. [9] and van Dijk et al. [3] focussed on understanding which characteristics define a lecturer as an ‘expert’, synthesising evidence from theoretical frameworks apart from students’ perspectives. Another work by Sutkins et al. identified different characteristics of the ‘good teacher’ in medical education [10]. However, we have witnessed a massive shift in students’ learning strategies in the last decades which strongly limits the applicability of previous evidence to more digitalised teaching contexts [11]. Among the studies mentioned in Sutkin’s review, only a few explored students’ preferences and needs, which should always be considered while designing effective lectures [4–6]. Moreover, none of these studies focussed on health professionals in rehabilitation (e.g., physiotherapists and speech therapists). These professionals need to gain high clinical knowledge and reasoning skills, compounded by a robust, practical apparatus. Thus, the transferability of previous findings to lecturers in the rehabilitation field may be limited due to differences in students’ required skills and knowledge. Finally, while the abovementioned evidence has been found in extra-European countries, there remains a lack of evidence from Mediterranean regions where a wider educational gap exists [12].

Qualitative studies aim at gaining relevant information about people’s personal life experiences [13] and are a crucial tool to understand students’ perceptions of lecturers’ characteristics that can facilitate their learning process. In line with that, the present qualitative study investigated the lecturers’ characteristics that facilitate students’ learning process in the rehabilitation sciences starting from Italian healthcare professional students’ voices. By doing so, we will widen the body of evidence in the educational field of rehabilitation, improving the future healthcare professionals’ learning process.

## Methods

### Study design

We performed a qualitative interview study. It was conducted in respect of the ‘Declaration of Helsinki’ and reported following the ‘Consolidated Criteria for Reporting Qualitative Research’ (COREQ) [14]. Ethical approval was obtained from the ethics committee of the Department of Human Sciences, University of Verona (Verona, Italy; approval date: 17/12/2020; 2020_31).

### Participants

Recruitment of study participants was done through purposive sampling to identify those most likely to yield valuable and appropriate information to answer our research question [15]. Specifically, we enrolled students attending the 2nd year of the Master of Science (MSc) degree in ‘Rehabilitation Sciences of Healthcare Professions’. This MSc is characterised by an interdisciplinary population of students who attended a Bachelor of Science (BSc) in rehabilitation according to the Italian register of rehabilitation healthcare professionals (i.e., physiotherapists, speech therapists, occupational therapists, orthoptists, podiatrists, psychiatric rehabilitation technicians, psychomotor therapists, and professional educators). Therefore, we invited students from this MSc as one of its main aims is to provide students with advanced skills in teaching. We considered students attending this MSc more appropriate than those following a BSc in the rehabilitation sciences due to their interest in the didactics subjects. Moreover, we chose students from the 2nd rather than 1st year as they had already attended different courses on self-reflection and qualitative research.

The MSc course leader (LF) was approached to contact the students. LF sent an email to the entire classroom explaining the aim of the study, the interview process, and the data confidentiality and anonymity. In the email, students were invited to contact VP directly if they were willing to partake in the study without answering the email sent by the course leader so that the latter did not know which students attended the interviews. This was done to avoid any form of dependability towards the course leader. Once VP was contacted, she collected the informed consent and arranged the interview. All the students were free to join the research and withdraw from it anytime. VP is a psychiatric rehabilitation technician, and LF is a physiotherapist and the course leader of the MSc in ‘Rehabilitation Sciences of Healthcare Professions’. They both identify as women.

### Data collection method

A semi-structured interview guide was created by a VP, SB and LF (Table 1). SB is a physiotherapist and PhD candidate, and temporary lecturer in ‘Teaching Methodology for Healthcare Professionals’. SB is trained in qualitative methodologies, with proficiency in conducting qualitative studies [16, 17]. SB identifies as a man. The interview guide consisted of open questions exploring different topics related to the characteristics of the lecturer that can facilitate students’ learning process. At the beginning of each interview, the participants filled in the informed consent. They provided their demographic (i.e., age, the gender they most identified with, and job), which the interviewer registered on an electronic sheet. The semi-structured interviews were performed in December 2020 by VP and lasted approximately one hour each. The interviews were conducted online, videoconferencing, and only with the interviewee. None of the participants had close relationships with the interviewer. No repeat interviews were conducted. An audio-visual recording of each interview was produced, transcribed *verbatim* and analysed by SB, VP, BG and IC, as reported in the following subsection.

**Table 1:** Interview Guide

Questions
1) I would like to start this interview by asking you to tell me which methods you usually adopt to study. 1a) According to your opinion, which factors can influence your studying process? (In the sense of learning or / and memorising better) 1b) Which teaching methods and lecturer characteristics can facilitate your study?
2) Tell me about that time you met a particular lecturer that facilitated your learning process during your university course… 2a) What did impress you about them? 2b) What strategies did they use? 2c) Which strategy(ies) did you find particularly useful to learn the topics and the subject? 2d) Have some strategies motivated you to study, which you did not think would have been effective? 2e) What was vital for you in how the lecturer conducted the lesson to learn better and memorise? 2f) Have these lecturers met your expectation? Before knowing them, which characteristics do you think a good lecturer should have had generally? 2 g) What do you think if I mention frontal didactics? Do you think it is a valuable methodology? 2 h) What if I mention the interactive didactics, such as workgroup… How did you find them? 2i) Did you think the interactive didacts were helpful in good learning? What are the obstacles you found in doing them?
3) Let’s talk about ‘skills’. Starting from the experience you have just told me, what were the lecturer’s skills that, according to you, make the difference in the learning process? 3a) What if I mention technical skills such as the ability to programme effective modules? 3b) What if I mention relational skills, such as communicative skills? 3c) What skills come to your mind if I mention transversal skills, such as those not directly connected to the lecturer’s skills in class that can be applied everywhere, in all workplaces?
4) What difficulty did you meet in your course relative to the lecturer’s didactic modality? 4a) Has this difficulty been perceived as an obstacle, or did it help you somehow?
5) What did you expect but miss in your university course regarding lecturing?
6) If you could contribute to teaching quality, what suggestions or strategies would you propose?
7) Would you like to add anything else that came to your mind?

BG is a physiotherapist and a PhD student in ‘Neurosciences’. IC is a social psychologist with a PhD in ‘Migrations and intercultural processes’. IC is trained in qualitative methodologies, with proficiency in conducting qualitative studies [18]. IC identifies as a woman; BG identifies as a man. SB and IC provided the abovementioned authors with all the necessary training to analyse the interviews. Moreover, they thoroughly followed them during this process.

### Data analysis

Descriptive sample analysis was conducted to collect information about the gender, age, and professional role of the interviewees. We analysed our data with a ‘Thematic Analysis’ (TA) [19]. TA is an independent qualitative descriptive approach described as “a method for developing, analysing and interpreting patterns across a qualitative dataset, which involves systematic processes of data coding to develop themes” [19]. Precisely, we followed the principles of Braun’s and Clarke’s ‘Reflexive Thematic Analysis’ (RTA) among the different approaches to TA (i.e., coding reliability TA, codebook approaches to TA, and RTA). RTA is an interpretive approach to qualitative data analysis “that facilitates the identification and analysis of patterns or themes in a given data set” [20]. RTA is situated in a ‘Big Q’ qualitative paradigm that involves qualitative data and methods whose qualitative values framework is characterised by adhering to a non-(post)positivist paradigm [21]. Therefore, RTA refused different practices considered relevant in other qualitative research paradigms (e.g., consensus coding, inter-coder reliability, data saturation etc.). These practices are infused “with assumptions about the nature of reality and meaningful knowledge” that follow a ‘small q’ (postpositivist) paradigm [19, 22]. Moreover, the researchers’ active and creative role in interpreting codes and themes and identifying those more relevant to the research question is fundamental rather than being a source of bias [23]. Since the analysis involved more than one researcher, the approach was as collaborative and reflexive as possible, intending to achieve richer interpretations [23].

However, it is fundamental to state our theoretical assumptions as researchers as our reflections are built upon them. For our study, we adopted an experiential qualitative framework because we illustrated the characteristics of the lecturer that facilitate students’ learning process to reflect the perception of social reality (rehabilitation healthcare professionals) [19, 24]. We adhered to a constructionist epistemology as themes’ meaning and meaningfulness were considered more important than their recurrency to answer our research question [24]. The use of thematic analysis in this study was majorly inductive, as we took the dataset as the starting point for our data analysis [19]. Thus, the data were not coded according to a pre-existing coding framework (i.e., the codebook of the deductive approach) [24]. However, it is worth noticing the impossibility of conducting an exclusively inductive analysis as one approach tends to predominate over the other [25] without being exclusive. Finally, the data coding was mostly semantic as we mostly stayed on the explicit or surface meanings of the data [19]. However, we tried to go beyond these descriptive levels of the data when possible. Thus, having clarified the theoretical assumptions and the choice of using RTA, the six steps of the RTA [23] were followed for the interview analysis (see Table 2).

**Table 2:** Steps of the ‘Reflexive Thematic Analysis’

Phases	Process	Authors’ Involvement	Authors’ Actions
1) Data familiarisation	All authors read and reread several times the transcriptions of the interviews. This process is fundamental to getting in contact with the data and taking notes of any impressions and insights.	All authors engaged in this phase, and they met to reflect upon their first insights	- Document theoretical and reflective thoughts: VP documented field notes (“Memos” and diary) during and after each interview to promote reflexivity. - Keep records of all data field notes, transcripts, and reflexive diary - Prolong engagement with data and triangulate different data collection modes to increase the probability that the research findings and interpretations will be found credible: all authors read and reread the data (transcripts of the interviews, memos and reflexive diary)
2) Coding	In this phase, two authors systematically coded the data through an open, evolving and organic process.	VP, GB and IC systematically coded the data. They adopted semantic data coding.	- Peer debriefing: memos were shared during research meetings for reflexive thoughts. - Audit trail of code generation: VP, BG, and IC coded data through the entire data set to identify interesting aspects in the data items that may form the basis of themes across the data set. - Documentation of all team meetings and peer debriefings to help researchers examine how their thoughts and ideas evolve as they engage more deeply with the data
3) Generating initial themes	The researchers generated initial themes from the codes, clustering similar or related codes.	VP, GB and IC generated initial themes separately, clustering similar codes together.	- Diagramming to make sense of theme connections: VP, BG and IC generated initial themes through inductive thematic analysis.
4) Reviewing and refining themes	The researcher reviewed the initial themes, reworking or discarding some until finding a final set of themes fitting the data.	All authors reviewed the coding and initial themes separately and then jointly and generated four themes that fit the data the most. VP, BG and IC reviewed the agreed themes against the codes and the entire dataset.	- Themes vetted by team members: the research team frequently met to refine the themes and clearly show how each theme was generated from the data.
5) Defining and naming themes	Finalising theme names and their definition develop the ‘story’ of each theme.	All authors finalised the final themes and definitions to set the basis of the written report.	- Peer debriefing and team consensus on themes: the research team met until the final themes were reached. - Documentation of theme naming.
6) Producing the report	The authors produced the final report and refined them if necessary.	VP, BG and IC selected the illustrative quotations from the interviews, and all authors reviewed and agreed. SB led the writing of the paper, and all authors participated in this phase.	- Producing the report using direct quotes from participants. - Report on reasons for theoretical, methodological, and analytical choices throughout the entire study.

## Results

Thirteen out of twenty students (age (mean and standard deviation): 27.8 ± 6.3, the gender they identified with (percentage and frequency): 15% Men N = 2, 85% Women N = 11) attending the 2nd year of the MSc in ‘Rehabilitation Sciences of Healthcare Professions’ (University of Verona, Verona, Italy) agreed to participate in the interviews. Among the participants, six were physiotherapists (46%), six were speech therapists (46%), and one was a psychiatric rehabilitation technician (8%) (See Table 3 for descriptive statistics of the enrolled participants).

**Table 3:** Participants’ characteristics

Participant ID	Age	Gender	Job
**P1** **P2**	34 26	W M	PT PT
**P3** **P4**	23 46	W M	ST PT
**P5** **P6**	24 25	W W	ST ST
**P7** **P8** **P9** **P10** **P11**	24 23 31 26 28	W W W W W	ST PT PT PT ST
**P12**	25	W	PRT
**P13**	26	W	ST

Legend: P, participant; W, woman; M, man; PT, physiotherapist; ST, speech therapist; PRT, psychiatric rehabilitation technician

From the analysis of the interviews, five themes were developed, and the most relevant quotations were reported in the next themes’ subsections. To indicate which participants made each statement, we reported their ID, gender, and speciality (according to Table 3) at the end of each quotation. These themes suggested the characteristics of lecturers in the rehabilitation sciences that can facilitate students’ learning process: 1) ‘A Performer who Interacts with the Classroom’, 2) A Flexible Planner who Adopts Innovative Teaching Skills’, 3) ‘A Motivator who Embraces Transformational Leadership’, 4) ‘A Facilitator Who Encourages a Constructive Learning Context’ and 5) ‘A Coach who Devises Strategies to Reach Shared Learning Goals’.

### Theme 1: a performer who interacts with the classroom

Some lecturers’ characteristics identified by the students made the authors generate the theme ‘A Performer who Interacts with the Classroom’ as they should entertain the students and keep their attention alive during the lesson. This performance is possible if the teacher creates debates and stimulates students’ interactions and reflections.

“It is important to ask [the students] about different topics covered during the lessons and to open a sort of debate or to stimulate reflection. These moments allow the group to reflect and reinforce the key concepts, synthesising what has been said” (P6, woman, 25, speech therapist).

In addition, lecturers must get close to their audience, using strategies to reduce tension and achieve a good learning climate.

“Being able to use irony and make students laugh while discussing difficult topics, without trivialising them, can be helpful. Laughing about the same thing can bring students closer” (P3, woman, 23, speech therapist).

Another relevant characteristic was the appropriate use of prosody (i.e., suprasegmental elements of the speech, such as intonation, stress, and rhythm). Lecturers should adapt their intonation, rhythm and tone of voice to the interlocutors and lecture topics to enhance attention and establish contact with the students, especially during a videotaped lesson where voice is the main communication channel.

“When they [lecturers] use [an effective] prosody, a speech constructed in a certain way that facilitates the attention of the listener, they can establish a better contact [with the students], conveying a[n effective] message too” (P6, woman, 25, speech therapist).

Another key feature is proxemics (i.e., human use of space). For students, lecturers who know how to manage the posture and the physical space of the classroom effectively convey a message, creating a physical link with the students.

“I think that a lecturer who moves, walks throughout the classroom, […] I feel they are more effective” (P6, woman, 25, speech therapist).

A performer who interacts with the class must also be able to adapt their language to the target audience in order to convey messages successfully.

“An important thing is the language. When they (lecturers) explain something, they should not take any technic terms for granted. They should use simple language, giving students the knowledge they need to understand that specific term” (P2, man, 26, physiotherapist).

However, according to the students, not only must the teacher be able to communicate with the class, they also must know how to organise the lesson using innovative teaching skills. Therefore, the second theme was created: ‘A Flexible Planner who Adopts Innovative Teaching Skills’.

### Theme 2: a flexible planner who adopts innovative teaching skills

Some characteristics identified by the students led the authors to define the theme ‘A Flexible Planner who Adopts Innovative Teaching Skills’. Lectures need to structure and schedule a lesson, taking into account different breaks or moments dedicated to debates, helping students to maintain in high attention level.

“She (lecturer) used to split the lessons [with learning moments and pause]. She asked for attention for a certain period, and then she gave you a break […] this induced more attention” (P1, woman, 34, physiotherapist).

Furthermore, lecturers must be able to identify and create relevant learning materials to ease students’ learning process. Materials such as videos, schemes, texts, updated handouts, concise slides, and explanatory images become real and practical knowledge sources if lecturers elaborate on and contextualise them. They allow the student to follow the lesson best and set a solid base for improving learning and facilitating memorisation.

“Giving material, like a handout, written and elaborated by the lecturer themself is very helpful and effective” (P6, woman, 25, speech therapist).

For students, teachers need to possess the necessary skills to apply innovative teaching strategies, using state-of-the-art methods that engage the student.

“Activities such as role-playing, practical laboratory, text analysis, clinical case, working in small workgroups are fundamental strategies as students actively do something” (P6, woman, 25, speech therapist).

Finally, the interviewees highlighted the importance of lecturers adapting to unforeseen events and developing practical problem-solving skills to find a rapid solution in real-time.

“Problem-solving skills […], even adapting to sudden events, such as the computer that doesn’t work or the projector that doesn’t turn on…” (P8, woman, 23, physiotherapist).

The first two themes encapsulated the characteristics related to the relationship that the lecture establishes with students as well as the psycho-pedagogical skills required to implement innovative teaching. However, our interviewees reported the need to be motivated and followed by lecturers that adopt enlightened leadership. Therefore, we generated the third theme that describes the lecture as a ‘Motivator who Embraces Transformational Leadership’.

### Theme 3: a motivator who embraces transformational leadership

The third theme highlighted the characteristics of a ‘Motivator who Embraces Transformational Leadership’ because our interviewees reported that lecturers should follow a leadership characterised by specific characteristics and motivate them in their learning process. In their opinion, lecturers should empathise with the students, avoid conflict, and be passionate about what they do.

“When a lecturer is very passionate and succeeds in transmitting it, it can ignite a similar passion in you” (P12, woman, 25, psychiatric rehabilitation technician).

“Teachers should be a motivator to learn” (P6, woman, 25, speech therapist).

In the interviews, students expressed the need for available lectures, physically, emotionally and mentally connected to them. A motivator who embraces transformational leadership must be inclined to listen to their students’ needs actively.

“She [a precise lecturer] knew how to listen, she was a lecturer that got closer to you while maintaining her role” (P5, woman, 24, speech therapist).

Furthermore, participants emphasise that a motivator who embraces transformational leadership must know how to use their role and function to create a supportive and constructive learning environment.

“Being able not to put themself [lecturer] at the same level as the student, and so maintaining their role, but still finding alternative strategies to get closer to the students.” (S9, woman, 31, physiotherapist).

Finally, the teacher, as a motivator who embraces transformational leadership, must be able to recognise and make explicit their limitations.

“If you do not know to answer, you do not pretend the opposite, or you start to climb on the mirror just to answer” (P8, woman, 23, physiotherapist).

Our study found that not only must lecturers play a crucial role in guiding students’ learning, they must also act as facilitators who create an active and constructive learning environment. Thus, we generated the fourth theme of the lecturer as a ‘Facilitator Who Encourages a Constructive Learning Context’.

### Theme 4: a facilitator who encourages a constructive learning context

Our participants find crucial that the lecturers facilitate a constructive dialogue with them, becoming ‘A Facilitator Who Encourages a Constructive Learning Context’ using each individual’s experiences as a critical resource to activate the learning process.

“I appreciate lecturers that are open to confrontation, to listen to students’ experiences” (P12, woman, 25, psychiatric rehabilitation technician).

“They (lecturer) gave straightforward examples that were also contextualised in our health sector” (P11, woman, 28, speech therapist).

For students, it also seems crucial that the lecturer, playing the role of facilitator, knows how to create a constructive learning environment, encourage dialogue, ask for feedback and adapt the language to the context.

“The lecturer that makes questions all the time […] By doing so, you have done a first round of study/review at the end of the lesson. Constant ongoing monitoring based on questions […] is also a nice way to catch up with the subject. Students can answer the lecturers’ questions, and the lecturer repeats or recaps the concepts using the words of the students” (P10, woman, 26, physiotherapist).

Finally, participants reported that they need someone who can help them to achieve their learning goals. This person should have a clear vision of what needs to be learned but also be able to negotiate these learning outcomes by involving the students in the final decision-making process. Thus, the last generated theme was ‘A Coach who Devises Strategies to Reach Shared Learning Goals’.

### Themes 5: a coach who devises strategies to reach shared learning goals

For students, the lecture must be able to actively involve students in the process of co-construction of knowledge and purpose, becoming a ‘Coach who Devises Strategies to Reach Shared Learning Goals’. In this way, the student feels more involved in the learning process.

“Lecturers should facilitate the students to understand where they are and where they are going, and so to share a common programme” (P6, woman, 25, speech therapist).

As coaches, they must also harness the knowledge students possess to co-construct theory with them.

“I appreciated a lot the lecturers who ask about our personal and working background, and the lecturers who can personalise the subjects based on who we are and our background” (P11, woman, 28, speech therapist).

Finally, they must be able to listen to their students and their interests, thus shaping the lesson and the content to be covered. In this way, it favours an active and effective learning process.

“Asking us what subjects we would like to go into detail, from which context we come from, what we would like to do, in my opinion, this is a thing that works a lot” (P2, man, 26, physiotherapist).

## Discussions

Starting from students’ perception, the present study investigated the characteristics of the lecturer in rehabilitation sciences that facilitate students’ learning process. In light of the above, the lecturer needs to possess the characteristics of a (1) ‘A Performer who Interacts with the Classroom’, (2) ‘A Flexible Planner who Adopts Innovative Teaching Skills’, (3) ‘A Motivator who Embraces Transformational Leadership’, (4) ‘A Facilitator Who Encourages a Constructive Learning Context’ and (5) ‘A Coach who Devises Strategies to Reach Shared Learning Goals’.

The interviewees reported specific characteristics of the lecturer that reflected a ‘Performer who Interacts with the Classroom’. Based on students’ words, lecturers in the rehabilitation sciences should learn different public-speaking strategies to keep students’ attention and create enjoyable and exciting lessons. ‘Teaching’ was already conceptualised by Schechner as one of the seven functions of ‘Performance’ and ‘Entertainment’ [26]. Entertainment and teaching share similar characteristics. The former is characterised by three elements: the actor, audience and performance. By the same token, the latter is characterised by the presence of a lecturer, who plays the role of the actor, the students, who are now the audience, and the lesson, which is the transposition of the performance, in the classroom. By creating entertaining activities, the lecturer can tap into the so-called ‘Intrinsic-value’, one of the mechanisms behind ‘Relevance’ [27]. ‘Relevance’ is defined as “a personally meaningful connection to the individual” and one of the ways to make a lesson memorable and worth attending [27]. ‘Intrinsic-value’ is the value of performing a task because it is enjoyable and exciting, as in this case, following a lecture.

Moreover, as performers, the voice gains a central role while teaching. As reported by Evans and Savage, a solid vocal presence in the classroom builds self-confidence and positive interactions with students [28]. As for this study, also our interviewees highlighted the importance of voice. According to them, the lecturer should regulate the rhythm and the tone of the voice while teaching and use a simple and adaptable pitch for creating learning experiences that are relevant and scalable for all students. Together with the voice, the use of the body, with an appropriate gesture and posture, are vital elements to create a performance worth experiencing and can influence the teaching-learning process, whether positively or negatively [29]. Also in our study, the use of proxemics (i.e., human use of space) was pointed out to be relevant by the interviewed students. They brought to the forefront that the body of the lecturer, through proper gesticulation and cunning use of the physical space, can be an instrument to effectively convey and reinforce the message by creating a physical bond with the words.

From the interviewees’ voices, the lecturer also needs to master the use of different innovative active-learning tools and didactics methodologies, picturing a ‘Flexible Planner who Adopts Innovative Teaching Skills’. As adults, students bring their experience to the learning process. They look for a link between the new information they are introjecting, what they already know about that lesson and the possible applications of what they are learning to the ‘real’ world [27]. This approach can tap into the so-called ‘Utility-Value’ (UV), i.e., the value that each of us gives to a task as relevant and useful to achieve a personal goal, present or future [27]. Our interviewees considered using different didactics methodologies and instruments helpful in answering this pragmatic need. This variety of teaching-delivery modalities allows students to reach optimal educational outcomes [30–32]. Besides, by using different strategies, lecturers can engage with the students by considering their preferred learning styles (i.e., visual, auditory, kinaesthetic, procedural, or a combination thereof) [33].

Moreover, the students reported the need to have structured and scheduled lessons. The lesson contents, the objective, and how students are assessed must be consistent, in line with the ‘Constructive Alignment’ approach to didactics [34]. The basic assumption of this approach is that every course needs to be designed so that each learning activity and assessment task is aligned with the learning objectives [35]. Moreover, the interviewees reported that unforeseen events, such as technology issues, can arise during the lesson. Thus, they stressed that lecturers need to accept uncertainty, adapt to those events and develop practical problem-solving skills to find a rapid solution in real-time.

Then, the interviewees reported different characteristics that generated the theme ‘Motivator who Embraces Transformational Leadership’. A motivator inspires the students to go beyond their perceived capabilities by adopting transformational leadership principles based on intellectual stimulation, individualised consideration, inspirational motivation and idealised influence [36, 37]. Our interviewees wanted a passionate lecturer capable of being empathetic, creating relevant interactions with the students and respecting the different roles. Passion is a relevant motivating factor and a significant need for high-quality learning and teaching [38]. It can increase students’ learning potential by creating an effective learning environment based on excitement and action. As pointed out by Fried, passion is not a personal feature found only in some people. Passion is discoverable, teachable and reproducible [39].

Being empathetic is another essential characteristic highlighted by our interviewees. Not only did they want to be engaged mentally, but they also wanted an emotional engagement. Therefore, lecturers should avoid creating conflicts and be available and connected to their students through emotions while lecturing [40]. These strategies were stressed by our interviewees, that wanted to feel close and connected to their lecturers. This feeling is reported in the literature as ‘Relatedness’, an intrinsic need of human beings to be related to significant people in their life, including lecturers [41]. In self-determination theory, optimal human functioning is hypothesised to depend on satisfying three basic psychological needs: autonomy, competence, and relatedness [8]. Students who perceive that their basic psychological needs are met tend to be intrinsically motivated, seeking new learning challenges and opportunities even in the absence of external rewards.

The interviewees reported different characteristics of lecturers that recalled those of an ‘A Facilitator Who Encourages a Constructive Learning Context’. The facilitator enables the shift from a lecturer-centred approach to a student-centred one, creating a positive environment for learning without giving just a flow of concepts [42]. As stated by our interviewees, the lecturer is called to develop long-term relationships with the single student based on interpersonal sharing. These attitudes establish a bidirectional learning process [40]. Moreover, our interviewees reported that lecturers should look for deeper interaction with the class, working hard to gain students’ trust. Numerous studies show that building solid lecturer-student relationships correlates with positive student outcomes, such as increased student engagement, learning and motivation [8]. Many interviewees highlighted their preference for a lecturer curious about their experience, especially when the class had worker students. This exchange is also helpful for the lecturer to give relevant examples tailored to students’ characteristics and experiences. But the confrontation cannot be intended just between the lecturers and the class. The interviewees reported the importance of peers’ interchange in workgroups. This interaction becomes stimulating once supported by the lecturers’ experiential wisdom [7].

To enhance the interaction between students and lecturers, feedback throughout the course plays a central role in the active-learning process [7]. Our interviewees stressed that not only should lecturers focus on the ‘summative’ evaluation, but they should also focus on ‘formative’ assessment [43]. The former is interested in exploring if the students reached the objective of the course at the end of it through an exam. Therefore, it cannot help students modify their learning process while attending the course but only after it. Instead, the latter assesses the learning process through feedback and itinerary exams throughout the module before the exam [43]. The formative assessment is fundamental since it informs the students about where they are in relation to the learning goals and what can be done to improve subsequent performances before the summative evaluation [43, 44].

Lastly, the interviewees reported that lecturers need to own the characteristics of a ‘Coach who Devises Strategies to Reach Shared Learning Goals’ to facilitate their learning process. Coaches in teaching lead their students towards mutually agreed objectives through ad hoc teaching and learning strategies that aim at reaching those objectives [45]. The coaches do not impose a strategy or content but always create a new method to support the class [46]. They can orientate their teaching decision process by stipulating the ‘learning contract’ [47]. Our interviewees pinpointed that this contract is fundamental to understanding the rationale of the course and agreeing with the students on the programme, the didactics methodologies and assessment modalities. According to Frank and Shariff, the ‘learning contract’ is also helpful in motivating and enhancing the students’ performance [48].

It is worth noticing that all the generated themes had to do more with the emotional and social spheres of learning. Our interviewees focussed on soft skills that lecturers need to have to facilitate the learning process rather than the knowledge of the subject. In their review, Sutkins et al. already noticed that many of the characteristics of the ‘good’ lecturer were ‘non-cognitive’ (e.g., relationship skills, emotional states etc.) rather than ‘cognitive’ (e.g., medical and technical knowledge) [10]. These results suggest that faculty development programmes should focus more on improving soft skills among lecturers as they usually receive less attention [10].

A limitation of this study is that only a few rehabilitation healthcare professionals (i.e., physiotherapists, speech therapists and psychiatric rehabilitation technicians) were reached. Moreover, most of them were white women. Finally, all the interviewees lived in a similar geographical area (i.e., northern Italy). This is particularly important to highlight since meanings attached to education might be influenced by gender, ethnicity and area of living. That notwithstanding, it is fundamental to hear the voice of these students as they have the potential to shed some light upon ways to improve education in Mediterranean countries. However, future studies should consider other ways to account for more diverse participants.

To conclude, the results of this study bring to the forefront some of the characteristics and skills that lecturers can develop to facilitate the students’ learning process. These skills are not strictly related to the contents per se but rather to how they are delivered and stem from a human sphere that other sources of knowledge cannot replace. We live in a world where information is readily available, and students can tap into different knowledge sources. To make a difference, lecturers need to develop specific skills to create the kind of lectures that are worth attending not only for their content but also for their value in human experience.

## Data Availability

Data are available upon reasonable request to the corresponding author.

## Abbreviations

COREQ: Consolidated criteria for reporting qualitative research
BSc: Bachelor of Science
MSc: Master of Science
(R)TA: Reflexive Thematic Analysis
P: participant
W: Woman/Women
M: Man/Men
PT: physiotherapist
ST: speech therapist
PRT: psychiatric rehabilitation technician
UV: Utility-Value
